# SQLE, A Key Enzyme in Cholesterol Metabolism, Correlates With Tumor Immune Infiltration and Immunotherapy Outcome of Pancreatic Adenocarcinoma

**DOI:** 10.3389/fimmu.2022.864244

**Published:** 2022-05-26

**Authors:** Weiqiang You, Jia Ke, Yufeng Chen, Zerong Cai, Ze-ping Huang, Peishan Hu, Xiaojian Wu

**Affiliations:** Department of Colorectal Surgery, Guangdong Provincial Key Laboratory of Colorectal and Pelvic Floor Diseases, Guangdong Institute of Gastroenterology, The Sixth Affiliated Hospital, Sun Yat-sen University, Guangzhou, China

**Keywords:** pancreatic adenocarcinoma, SQLE, prognosis, miRNA, tumor immune infiltration, immunotherapy

## Abstract

**Background:**

Pancreatic adenocarcinoma (PAAD) is a treatment-refractory cancer with poor prognosis. Accumulating evidence suggests that squalene epoxidase (SQLE) plays a pivotal role in the development and progression of several cancer types in humans. However, the function and underlying mechanism of SQLE in PAAD remain unclear.

**Methods:**

SQLE expression data were downloaded from The Cancer Genome Atlas and the Genotype-Tissue Expression database. SQLE alterations were demonstrated based on the cBioPortal database. The upstream miRNAs regulating SQLE expression were predicted using starBase. The function of miRNA was validated by Western blotting and cell proliferation assay. The relationship between SQLE expression and biomarkers of the tumor immune microenvironment (TME) was analyzed using the TIMER and TISIDB databases. The correlation between SQLE and immunotherapy outcomes was assessed using Tumor Immune Dysfunction and Exclusion. The log-rank test was performed to compare prognosis between the high and low SQLE groups.

**Results:**

We demonstrated a potential oncogenic role of SQLE. SQLE expression was upregulated in PAAD, and it predicted poor disease-free survival (DFS) and overall survival (OS) in patients with PAAD. “Amplification” was the dominant type of SQLE alteration. In addition, this alteration was closely associated with the OS, disease-specific survival, DFS, and progression-free survival of patients with PAAD. Subsequently, hsa-miR-363-3p was recognized as a critical microRNA regulating SQLE expression and thereby influencing PAAD patient outcome. *In vitro* experiments suggested that miR-363-3p could knock down the expression of SQLE and inhibit the proliferation of PANC-1. SQLE was significantly associated with tumor immune cell infiltration, immune checkpoints (including PD-1 and CTLA-4), and biomarkers of the TME. KEGG and GO analyses indicated that cholesterol metabolism-associated RNA functions are implicated in the mechanisms of SQLE. SQLE was inversely associated with cytotoxic lymphocytes and predicted immunotherapy outcomes.

**Conclusions:**

Collectively, our results indicate that cholesterol metabolism-related overexpression of SQLE is strongly correlated with tumor immune infiltration and immunotherapy outcomes in patients with PAAD.

## Introduction

Pancreatic adenocarcinoma (PAAD) is currently one of the most aggressive and malignant tumors with a 5-year survival rate of only 10% (1, 2). It is the seventh leading cause of cancer-related death worldwide (3). Given the long asymptomatic disease progression and poor early detection, 80% of patients with PAAD have an advanced or metastatic stage at diagnosis, rendering a grim prognosis (4–6). In recent years, despite improvements in perioperative chemotherapy, radiotherapy techniques, immune checkpoint inhibitors, and comprehensive treatments, the number of deaths due to PAAD has been steadily increasing (7, 8). Immunotherapy has shown favorable prospects for the treatment of solid tumors, especially when combined with other targeted drugs (9). Although tumor mutational burden (TMB), microsatellite status, and programmed cell death-ligand 1 (PD-L1) expression have been used to predict the effect of immunotherapy (10, 11), the efficiency was limited in PAAD. Therefore, there is an urgent need to identify more effective therapeutic targets and develop new promising strategies for PAAD.

Cholesterol is the major sterol in mammalian cell membranes, maintaining cell integrity and fluidity and forming intracellular homeostasis (12). The biosynthetic pathway from acetyl-CoA to cholesterol involves nearly 30 enzymatic reactions, including the initial mevalonate (MVA) pathway, subsequent squalene biosynthesis, and ultimate sterol conversion (13–15). Squalene epoxidase (SQLE) is the second rate-limiting enzyme in cholesterol biosynthesis that catalyzes the conversion of squalene to 2,3-epoxysqualene (16, 17). SQLE promotes the initiation and progression of non-alcoholic steatohepatitis by regulating cholesterol metabolism (18). Notably, an increasing number of studies have shown that SQLE expression is closely correlated with the progression, invasion, and metastasis of multiple tumors, such as breast cancer (19), hepatocellular carcinoma (20), esophageal cancer (21), prostate cancer (22), colorectal cancer (23), and lung cancer (24). In addition, the inhibitor terbinafine, which targets SQLE, showed efficient tumor suppression and represents a new strategy for solid tumor treatment (25). Recent research has emphasized that the glycolysis-cholesterol synthesis axis affects the outcome and prognosis of PAAD (26). However, a comprehensive analysis, including the expression, prognosis, and mechanism of SQLE in PAAD, has not yet been conducted. Additionally, the relationship between SQLE and the tumor immune microenvironment in PAAD remains unclear.

In this study, we first analyzed the expression level of SQLE and its prognostic value in various types of human cancers, illustrating its potential oncogenic role. Subsequently, microRNAs (miRNAs) were determined to be vital regulators of SQLE and to influence the outcome of patients with PAAD. Our results confirmed that SQLE is significantly associated with tumor immune cell infiltration, immune checkpoints, and biomarkers of the tumor immune microenvironment. RNA functions associated with cholesterol metabolism were found to be implicated in the mechanisms of SQLE. Finally, a high SQLE level was indicative of a poor immunotherapy effect in melanoma and PAAD. Together, cholesterol metabolism-related overexpression of SQLE is strongly correlated with poor prognosis, tumor immune infiltration, and immunotherapy outcomes in PAAD.

## Materials and Methods

### Cell Culture

The human PDAC cell line PANC-1 was obtained from the American Type Culture Collection (ATCC) and cultured in DMEM medium (Gibco, Carlsbad, CA) supplemented with 10% FBS (Gibco, Carlsbad, CA) and 1% penicillin/streptomycin (Gibco, Carlsbad, CA) in 5% CO_2_ at 37°C.

### Western Blotting

Total proteins were extracted in RIPA buffer, and the protein concentration was measured by BCA Protein Assay Kit (Thermo Fisher Scientific, Waltham, MA). Protein was resolved in 10% Tris-SDS-PAGE gels and transferred to PVDF membranes (Millipore, Darmstadt, Germany). The membranes were incubated with anti-human SQLE antibody (Proteintech, Chicago, IL) at a dilution of 1:1000 and then probed with HRP-conjugated secondary antibody (Proteintech, Chicago, IL).

### Cell Transfection

The miR-363-3p mimics (Genepharma, Shanghai, China) or negative control (NC) was transfected into PANC-1 cells using Lipofectamine 3000 Transfection Reagent (Invitrogen, Karlsruhe, Germany) according to the manufacturer’s instructions.

### Cell Viability

Cells were plated in 96-well plates at a density of 2000 cells per well. Cell viability was assessed using CCK-8 (Gaithersburg, MD). OD450 values were determined on Day 0, 1, 2, 3, and 4.

### Correlation Between SQLE Expression and Immune Cell Infiltration

TIMER (27) and TIMER2.0 (28) were used as servers for the comprehensive analysis of SQLE expression in 33 types of human cancer, infiltration of tumor immune cells, and the expression of immune checkpoints in PAAD. EPIC (29) and McP-Counter (30) were used to validate the immune cell infiltration from SQLE expression profiles. One-way ANOVA was used to test the significant differences. Statistical significance was set at p < 0.05.

### GEPIA Database Analysis

GEPIA (31) is a web tool for gene expression analyses based on The Cancer Genome Atlas (TCGA) and Genotype-Tissue Expression databases. We used GEPIA to analyze SQLE expression in 10 types of human cancers, namely, ACC, DLBC, LAML, LGG, OV, PAAD, SKCM, TGCT, THYM, and UCS. Statistical significance was set at p < 0.05. We also conducted survival analyses for SQLE, including overall survival (OS) and disease-free survival (DFS). The correlation of SQLE with ACAT2, HMGCR, HMGCS1, IDI1, and LDLR in PAAD and pan-cancer was analyzed, and the top 100 SQLE-correlated genes were identified using GEPIA.

### StarBase Database Analysis

The Starbase database (32) was first used to predict the miRNAs upstream of SQLE. PITA, RNA22, miRmap, microT, miRanda, PicTar, and TargetScan were used to identify miRNAs binding to SQLE, and miRNAs that were present in more than two programs were included in further analyses. We also used starBase to perform miRNA expression and correlation analyses for miRNA and SQLE in PAAD.

### Kaplan-Meier Plotter Analysis

Kaplan-Meier plotter (33), a database evaluating the genes or miRNAs that are associated with survival in human cancer types, including PAAD, was used to perform survival analysis for miRNAs in PAAD. A log-rank p < 0.05 was defined as statistically significant.

### Genetic Alteration Analysis

cBioPortal web (34, 35) was used to analyze the alteration frequency, mutation type, and copy number alteration of SQLE in human cancers. The mutated site information of SQLE is displayed in a schematic diagram of the protein structure. The prognostic value of SQLE alterations, including OS, disease-specific survival (DSS), DFS, and progression-free survival (PFS), was determined using survival analysis. In addition, immunohistochemical images of SQLE in tumor and normal tissues were obtained. Log-rank p-values were also generated.

### TISIDB Database Analysis

TISIDB is a web portal for tumor and immune system interaction (36). The relationship between SQLE expression and tumor immune biomarkers in PAAD, including lymphocytes, MHC molecules, immune inhibitors, and immunostimulators, was analyzed using TISIDB. The p-value and Spearman’s correlation coefficients (rho) were calculated automatically.

### Gene Enrichment Analysis

STRING website (37) was used to determine the SQLE-binding proteins network. Kyoto Encyclopedia of Genes and Genomes (KEGG) pathway analysis was performed by “hiplot” (unpublished, https://hiplot.com.cn), which is a free and comprehensive cloud platform for scientific computation and visualization. Gene ontology (GO) analyses, including biological process (BP), cellular component (CC), and molecular function (MF), were obtained from the DAVID database (https://david.ncifcrf.gov). A two-tailed p < 0.05 was considered statistically significant.

### Statistical Analysis

Data are shown as the mean ± standard deviation of at least three independent experiments. Kaplan-Meier survival analysis was used to compare survival times with the log-rank test. Spearman’s correlation coefficient was used to determine the relationship between the two variables. Statistical significance was set at p < 0.05.

## Results

### SQLE Expression in the Pan-Cancer Analysis

**Figure 1:**
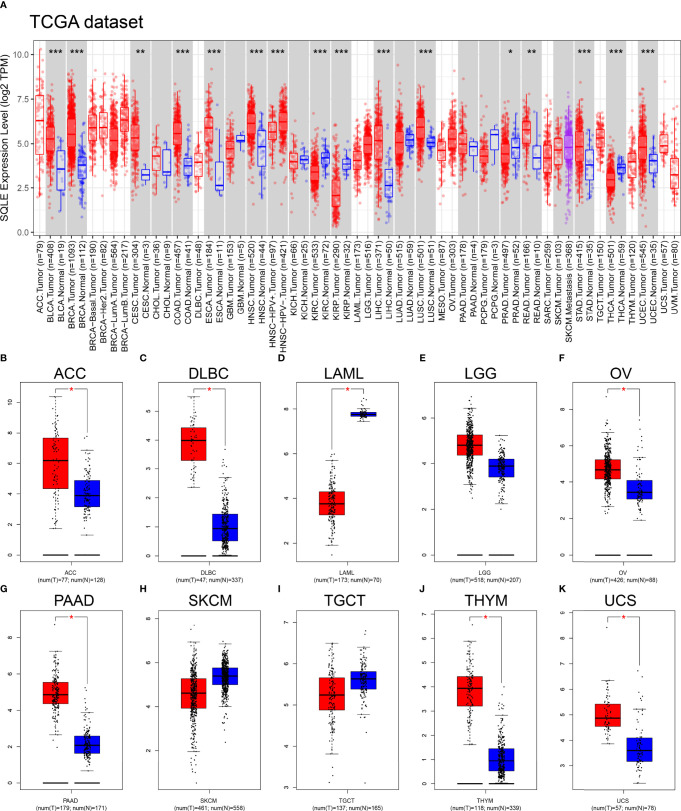
SQLE expression level in human cancers. **(A)** SQLE expression in 33 types of human cancer based on TCGA dataset. **(B–K)** SQLE expression in ACC **(B)**, DLBC **(C)**, LAML **(D)**, LGG **(E)**, OV **(F)**, PAAD **(G)**, SKCM **(H)**, TGCT **(I)**, THYM **(J)**, and UCS **(K)** compared with corresponding TCGA and GTEx normal tissues. *p < 0.05; **p < 0.01; ***p < 0.001.

We first explored SQLE expression levels in 33 types of human cancers based on TCGA dataset. As shown in **Figure 1A**, SQLE expression was significantly higher in tumors than in normal tissues in BLCA, BRCA, CESC, COAD, ESCA, HNSC, LIHC, LUSC, READ, STAD, and UCEC. SQLE expression was distinctly downregulated in KIRC, KIRP, PRAD, and THCA cells. Owing to an insufficient number of normal tissues as controls for several cancer types in the TCGA dataset, we verified the difference in SQLE expression between normal and tumor tissues in 10 types of human cancers by including normal tissue from the GTEx consortium of the GEPIA database. SQLE expression level was prominently increased in ACC, DLBC, OV, PAAD, THYM, and UCS and was dramatically downregulated in LAML. However, no significant differences were observed in LGG, SKCM, and TGCT (**Figures 1B–K**). Notably, SQLE expression in PAAD was markedly upregulated when an adequate number of normal tissues were used as controls. In summary, aberrant SQLE expression was observed in 22 types of human cancers, implying that SQLE has a tumorigenic function.

### SQLE Is Associated With the Prognosis of Multiple Human Cancer

**Figure 2:**
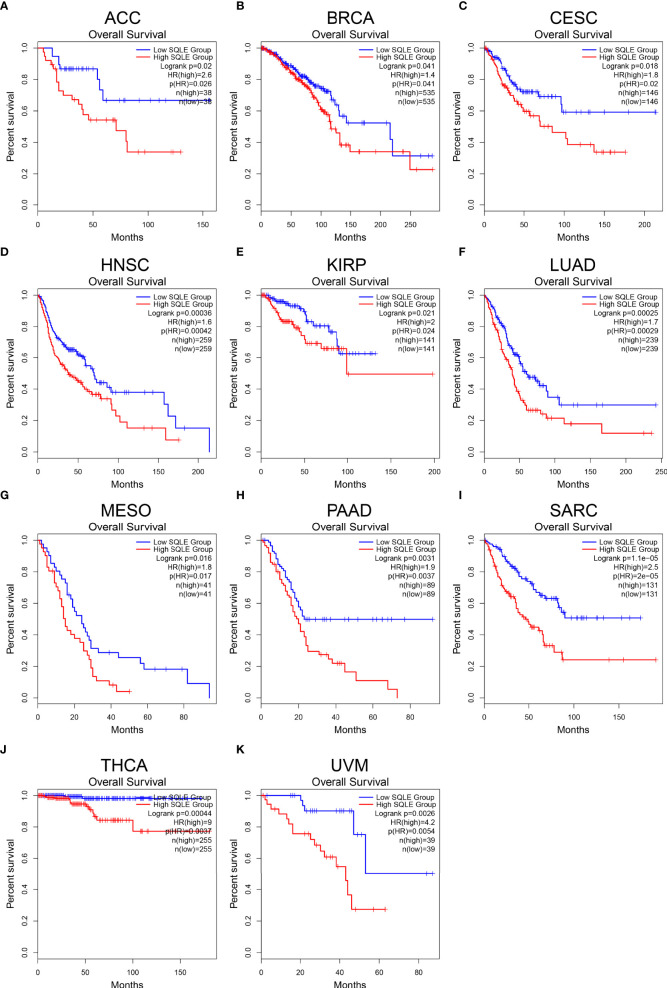
Overall survival (OS) analysis for SQLE in multiple human cancers. **(A–K)** The OS curves of SQLE in ACC **(A)**, BRCA **(B)**, CESC **(C)**, HNSC **(D)**, KIRP **(E)**, LUAD **(F)**, MESO **(G)**, PAAD **(H)**, SARC **(I)**, THCA **(J)**, and UVM **(K)**.

To further reveal the significance of SQLE in tumors, we performed survival analyses, including OS and DFS, in 33 types of human cancers. As shown in **Figure 2**, high SQLE expression predicted unfavorable OS in the following 11 cancer types: ACC (p = 0.02), BRCA (p = 0.041), CESC (p = 0.018), HNSC (p < 0.001), KIRP (p = 0.021), LUAD (p < 0.001), MESO (p = 0.016), PAAD (p = 0.0031), SARC (p < 0.001), THCA (p < 0.001), and UVM (p = 0.0026). Moreover, overexpression of SQLE was linked to poor DFS in ACC, BLCA, HNSC, LUSC, PAAD, SARC, and UVM (**Supplementary Figure S1**, p < 0.05). In other types of human cancers, there was no significant difference observed in SQLE expression between the high and low groups. Taken together, our results reveal that SQLE overexpression correlates with poor prognosis in patients with PAAD.

### Analysis of SQLE Alterations in PAAD

**Figure 3:**
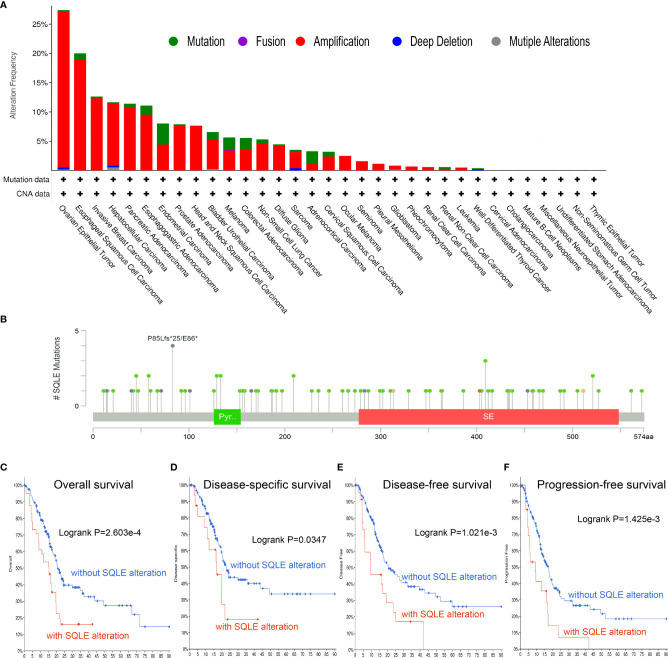
Mutation characteristics of SQLE determined using the cBioPortal database. **(A)** The alteration frequency with mutational types of SQLE. **(B)** The mutational sites of SQLE. **(C–F)** The correlation between SQLE and OS **(C)**, DSS **(D)**, DFS **(E)**, and PFS **(F)** in PAAD.

To investigate the frequency and category of SQLE mutations in human cancers, we conducted a gene alteration analysis. The highest alteration frequency of SQLE (> 25%) was observed in patients with ovarian epithelial tumors, with “amplification” as the dominant type (**Figure 3A**). Significantly, more than 10% of SQLE alterations (including “amplification” and “mutation”) were detected in PAAD patients (**Figure 3A**). Furthermore, we explored the location and number of SQLE alterations and found that the P85Lfs*25/E86* domain was detected in 4 cases, which was the most mutated location (**Figure 3B**). Additionally, the relationship between SQLE alterations and PAAD prognosis was demonstrated. Our results indicate that PAAD patients with SQLE alterations had worse OS (p = 2.603e-4), DSS (p = 0.0347), DFS (p = 1.021e-3), and PFS (p = 1.425e-3) than patients without SLQE alterations (**Figures 3C–F**). Together, SQLE alterations were frequently probed in PAAD and found associated with an unfavorable prognosis in patients with PAAD.

### Analysis of Upstream miRNAs Regulating SQLE in PAAD

**Figure 4:**
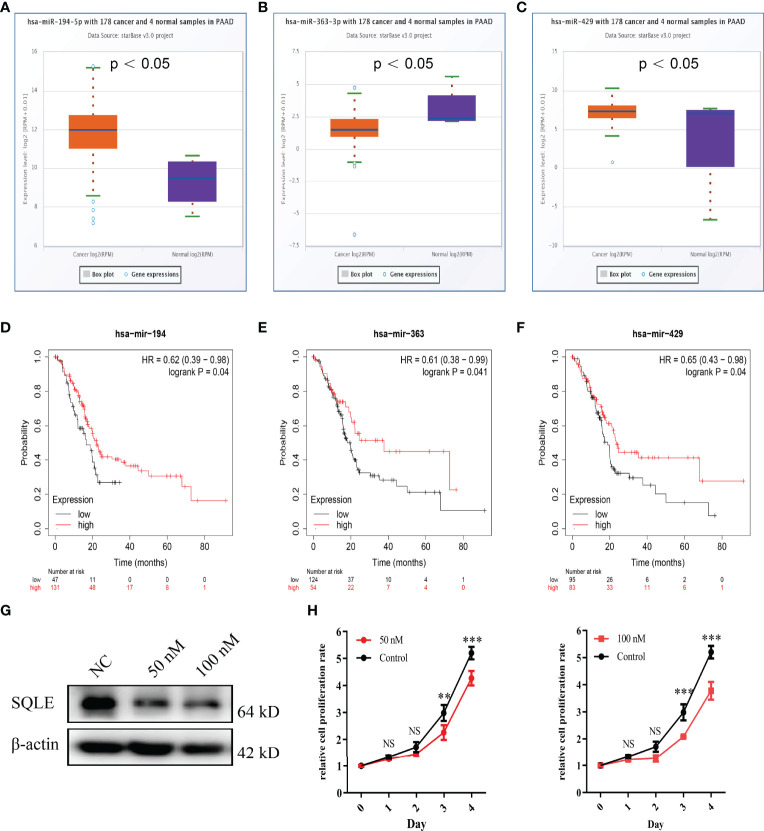
Identification of miRNAs as potential upstream regulators of SQLE in PAAD. **(A–C)** Expression of miR-194-5p **(A)**, miR-363-3p **(B)**, and miR-429 **(C)** in PAAD and control normal tissues. **(D–F)** The prognostic values of miR-194-5p **(D)**, miR-363-3p **(E)**, and miR-429 **(F)** in PAAD obtained using the Kaplan-Meier plotter. **(G)** The SQLE knockdown efficiency of 50 nM and 100 nM of miR-363-3p mimics on Day 2. **(H)** The effects of 50 nM and 100 nM of miR-363-3p mimics on *in vitro* proliferation in PANC-1. Two-way ANOVA test (n=3). **p value < 0.01; ***p value < 0.001; NS, no significance.

MicroRNAs (miRNAs) can bind to and regulate the expression of target genes. To identify the miRNAs that regulate SQLE expression, we analyzed the upstream miRNAs that could potentially target SQLE. We found 21 miRNAs that could be responsible for regulating SQLE expression in the pan-cancer analysis (**Table 1**). Next, we focused on these miRNAs in PAAD. As shown in **Figures 4A–C**, the expression of hsa-miR-194-5p, hsa-miR-363-3p, and hsa-miR-429 was different in the tumor and normal tissues, and therefore these miRNAs were confirmed as vital regulatory molecules (p < 0.05). High expression of these three miRNAs predicted favorable OS in PAAD (**Figures 4D–F**, p < 0.05). This phenomenon was not observed for the other 18 miRNAs in PAAD. It is well known that miRNAs negatively regulate their target genes (38). As presented in **Table 1**, SQLE expression showed a negative correlation with hsa-miR-363-3p but a positive correlation with hsa-miR-194-5p and hsa-miR-429 in PAAD. Thus, we hypothesized that hsa-miR-363-3p is an upstream miRNA of SQLE.

**Table 1:** The expression correlation between predicted miRNAs and SQLE in PAAD analyzed by starBase database.

Gene	miRNA	R-value	P-value
SQLE	hsa-miR-584-5p	0.187	1.24E-02*
SQLE	hsa-miR-194-5p	0.216	3.77E-03*
SQLE	hsa-miR-579-3p	-0.011	8.84E-01
SQLE	hsa-miR-664b-3p	-0.040	5.95E-01
SQLE	hsa-miR-205-5p	0.049	5.17E-01
SQLE	hsa-miR-367-3p	-0.040	5.98E-01
SQLE	hsa-miR-363-3p	-0.189	1.17E-02*
SQLE	hsa-miR-25-3p	0.068	3.67E-01
SQLE	hsa-miR-92a-3p	0.123	1.02E-01
SQLE	hsa-miR-32-5p	-0.012	8.76E-01
SQLE	hsa-miR-92b-3p	0.048	5.24E-01
SQLE	hsa-miR-429	0.190	1.10E-02*
SQLE	hsa-miR-371a-5p	-0.093	2.19E-01
SQLE	hsa-miR-200c-3p	0.072	3.37E-01
SQLE	hsa-miR-200b-3p	0.230	1.97E-03*
SQLE	hsa-miR-495-3p	0.012	8.76E-01
SQLE	hsa-miR-133b	-0.143	5.69E-02
SQLE	hsa-miR-381-3p	-0.011	8.80E-01
SQLE	hsa-miR-495-3p	0.012	8.76E-01
SQLE	hsa-miR-133a-3p	-0.179	1.67E-02*
SQLE	hsa-miR-1298-5p	0.167	2.54E-02*

*p value < 0.05.

To explore the function of hsa-miR-363-3p in PAAD, we performed *in vitro* experiments using miR-363-3p mimic. Western blot results confirmed that both 50 nM and 100 nM mimics could effectively knock down the expression of SQLE in PANC-1 (**Figure 4G**). Subsequently, CCK8 results showed that the mimic could inhibit the proliferation ability of PANC-1 (**Figure 4H**). These results indicated that miR-363-3p could regulate the expression of SQLE and then inhibit cell proliferation in PAAD.

### SQLE Expression Was Closely Related to Immune Cell Infiltration in PAAD

**Figure 5:**
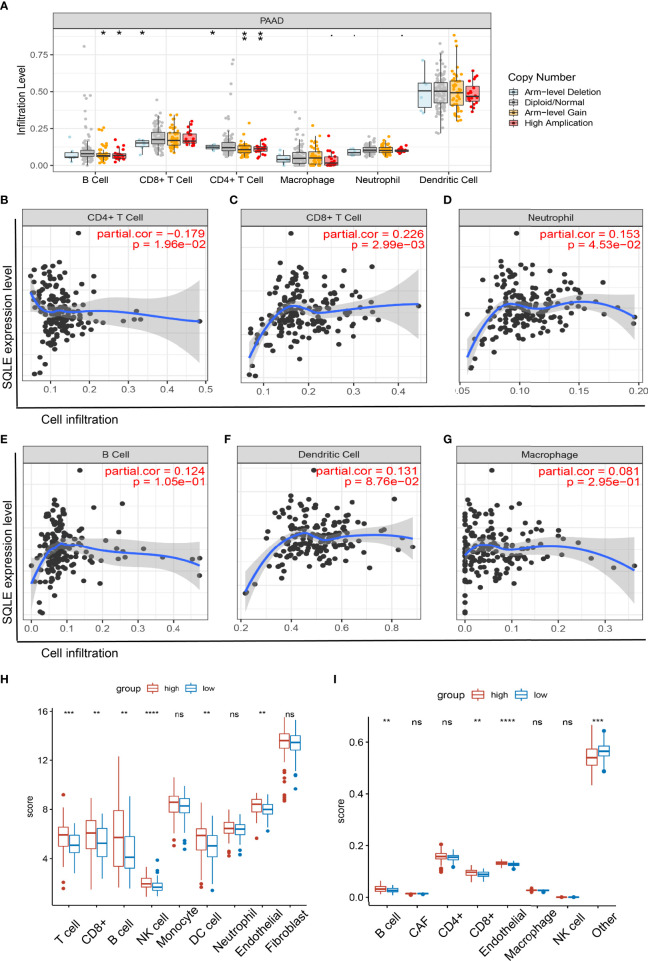
Relationship of immune cell infiltration with SQLE expression in PAAD. **(A)** Infiltration of immune cells under different alterations of SQLE in PAAD. **(B–G)** Correlation of SQLE with CD4+ T cells **(B)**, CD8+ T cells **(C)**, neutrophils **(D)**, B cells **(E)**, dendritic cells **(F)**, and macrophages **(G)**. **(H, I)** Differences of immune-infiltrating cells between SQLE high (n = 89) and low (n = 89) groups performed by McP-Counter **(H)** and EPIC **(I)**. *p < 0.05, **p < 0.01, ***p < 0.001, and ****p < 0.0001. NS, no significance.

SQLE is a key enzyme in cholesterol metabolism and is involved in important lymphocyte functions (39, 40). Therefore, we explored the relationship between SQLE expression and immune cell infiltration in PAAD patients. The copy number of SQLE could affect the infiltration of B cells, CD8+ T cells, and CD4+ T cells (**Figure 5A**). In addition, SQLE expression was negatively correlated with CD4+ T cells (**Figure 5B**). In contrast, SQLE expression positively correlated with the infiltration of CD8+ T cells (**Figure 5C**) and neutrophils (**Figure 5D**). Our results also demonstrated that SQLE expression did not affect the infiltration of the other three types of immune cells: B cells, dendritic cells (DCs), and macrophages (**Figures 5E–G**).

Furthermore, these results were validated in 178 patients with PAAD from the TCGA cohort. Patients were divided into two groups according to the median expression level of SQLE. McP-Counter and EPIC methods were performed to validate the immune cell infiltration in 2 groups. The McP-Counter results showed that there were significant differences in T cell, CD8+ T cell, B cell, NK cell, DC, and endothelial cell (**Figure 5H**), which was consistent with EPIC results (**Figure 5I**). In conclusion, SQLE expression has a complex regulatory effect on immune cell infiltration in PAAD.

### Correlation of SQLE With Biomarkers of Tumor Immune Microenvironment

**Figure 6:**
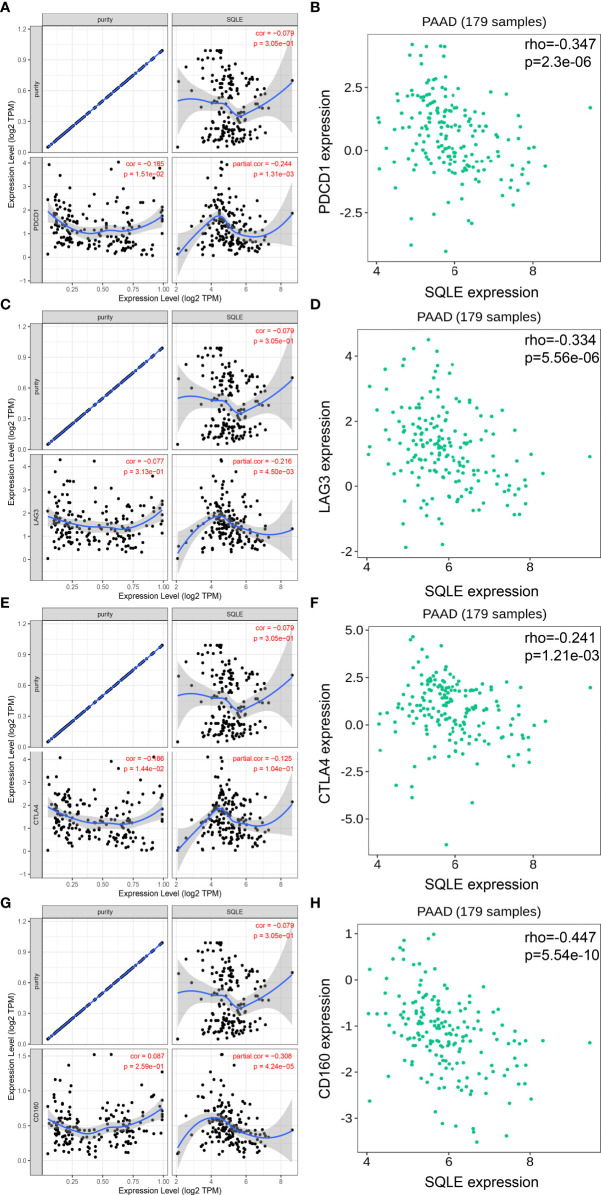
SQLE expression is closely related to PDCD1, LAG3, CTLA4, and CD160 expression in PAAD. **(A, B)** Spearman correlation of SQLE expression with PDCD1 expression in PAAD adjusted by purity in TIMER **(A)** and TISIDB **(B)**. **(C, D)** Spearman correlation of SQLE expression with LAG3 expression in PAAD adjusted by purity in TIMER **(C)** and TISIDB **(D)**. **(E, F)** Spearman correlation of SQLE expression with CTLA4 expression in PAAD adjusted by purity in TIMER **(E)** and TISIDB **(F)**. **(G, H)** Spearman correlation of SQLE expression with CD160 expression in PAAD adjusted by purity in TIMER **(G)** and TISIDB **(H)**.

To further investigate the relationship between SQLE and tumor immune biomarkers, we used the GEPIA and TISIDB databases. Our results showed that SQLE expression was significantly negatively correlated with PDCD1 (**Figure 6A**), LAG3 (**Figure 6C**), cytotoxic T-lymphocyte associated protein 4 (CTLA4, **Figure 6E**), and CD160 (**Figure 6G**)-all checkpoint inhibitors in the GEPIA database analysis. Our TISIDB database analysis confirmed these findings. SQLE expression was related to PDCD1 (**Figure 6B**, rho = -0.347, p < 0.01), LAG3 (**Figure 6D**, rho = -0.334, p < 0.01), CTLA4 (**Figure 6F**, rho = -0.241, p < 0.01), and CD160 (**Figure 6H**, rho = -0.447, p < 0.01). We performed further analyses to reveal the correlation among SQLE expression, copy number, methylation, and tumor immune features in PAAD, including lymphocytes (**Supplementary Figure S2-A**), immuno-inhibitors (**Supplementary Figure S2-B**), MHC molecules (**Supplementary Figure S2-C**), and immunostimulators (**Supplementary Figure S2-D**). Remarkably, the relationship between SQLE expression and tumor immune features was always contrary to the results of SQLE methylation (**Supplementary Figure S2**). Our results indicate that SQLE might function as a regulator of the immune microenvironment in PAAD.

### Enrichment Analysis of SQLE

**Figure 7:**
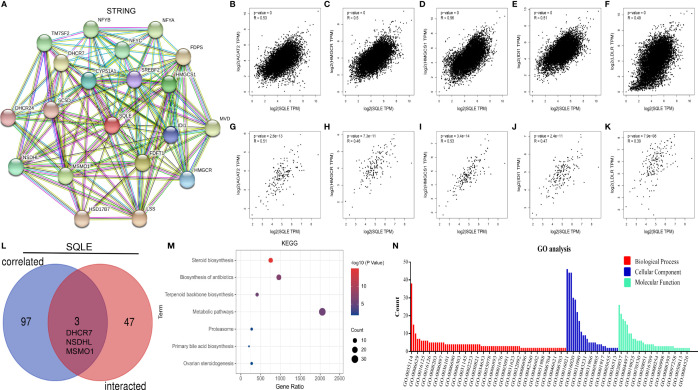
SQLE-related gene enrichment analysis. **(A)** The top 20 SQLE-binding proteins using the STRING tool. **(B–F)** The expression correlation between SQLE and the targeting genes ACAT2 **(B)**, HMGCR **(C)**, HMGCS1 **(D)**, IDI1 **(E)**, and LDLR **(F)** in human cancers. **(G–K)** The expression correlation between SQLE and the targeting genes ACAT2 **(G)**, HMGCR **(H)**, HMGCS1 **(I)**, IDI1 **(J)**, and LDLR **(K)** in PAAD. **(L)** Interaction analysis of the SQLE-binding and related genes. **(M)** KEGG pathway analysis of the SQLE-binding and interacting genes. **(N)** GO analysis for the molecular function of the SQLE-binding and interacting genes.

SQLE is a pivotal gene regulating cholesterol biosynthesis. Therefore, we performed an enrichment analysis of SQLE-related partners. A list of 50 SQLE-binding proteins was obtained from the STRING database. We constructed a network of 20 proteins that were most strongly associated (**Figure 7A**), and most of these proteins were involved in cholesterol metabolism. The top 100 genes related to SQLE expression pan-cancer were selected from the GEPIA2 database analysis. As presented in **Figures 7B–F**, SQLE expression level was positively correlated with ACAT2 (R = 0.53), HMGCR (R = 0.5), HMGCS1 (R = 0.56), IDI1 (R = 0.51), and LDLR (R = 0.49) genes (all p < 0.001). We obtained similar results for PAAD (**Figures 7G–K**, all p < 0.001). A combined analysis of the two datasets suggested three common molecules, namely, DHCR7, NSDHL, and MSMO1 (**Figure 7L**). Subsequently, we conducted KEGG and GO enrichment analyses. The results of the former showed that “metabolic pathways” and “steroid biosynthesis” were involved in the function of SQLE in carcinogenesis (**Figure 7M**). The results of the latter implied that these genes were related to oxidation-reduction, cholesterol biosynthesis, iron ion binding, and oxidoreductase activity, among other reactions (**Figure 7N**). The annotations of the X-axis in **Figure 7N** are presented in **Supplementary Table S1**.

### SQLE Is Associated With Immunotherapy Outcome of Cancer

**Figure 8:**
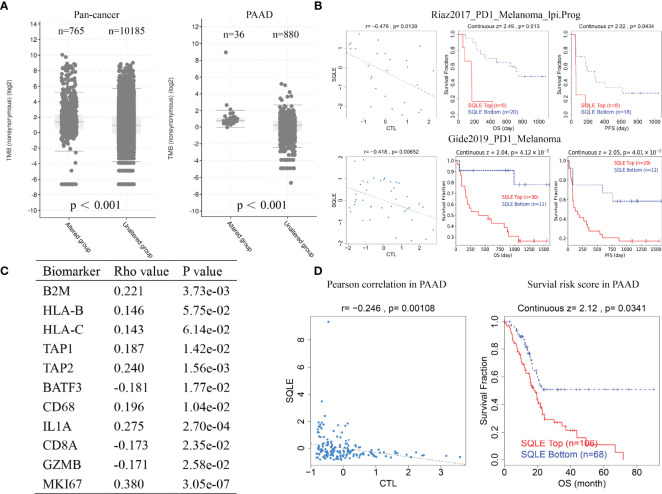
SQLE predicts poor outcome of immunotherapy in cancer. **(A)** The association of SQLE alteration and tumor mutational burden (TMB) pan-cancer (left) and in PAAD (right). The Wilcoxon test was used for statistical analyses. **(B)** The results of two clinical trials suggest that SQLE expression correlates with the unfavorable outcome of immunotherapy in melanoma. **(C)** Correlation between SQLE expression and biomarkers of MHC (B2M, HLA-B, HLA-C, TAP1, and TAP2), dendritic cells (BATF3), macrophages (CD68 and IL1A), type-I anti-tumor responses (CD8A and GZMB), and cell proliferation (MKI67) in 178 tumor tissues of PAAD. **(D)** SQLE correlates with the outcome of immunotherapy in PAAD.

TMB is a favorable predictor of immunotherapy. Our results suggest that SQLE alteration correlated with high TMB pan-cancer and in PAAD (**Figure 8A**, p < 0.001). We also analyzed the results of two clinical trials of anti-PD1 treatment in melanoma and found that SQLE expression was inversely associated with cytotoxic lymphocyte levels (CTLs), OS, and PFS in melanoma patients (**Figure 8B**, p < 0.05). Subsequently, we analyzed SQLE expression and its association with biomarkers of MHC (B2M, HLA-B, HLA-C, TAP1, and TAP2), dendritic cells (BATF3), macrophages (CD68 and IL1A), type-I anti-tumor responses (CD8A and GZMB), and cell proliferation (MKI67) in 178 PAAD tissues (**Figure 8C**). SQLE was positively associated with MHC molecules (B2M, r = 0.221; HLA-B, r = 0.146; HLA-C, r = 0.143; TAP1, r = 0.187; and TAP2, r = 0.240), macrophages (CD68, r = 0.196 and IL1A, r = 0.275), and cell proliferation (MKI67, r = 0.380) but negatively associated with dendritic cells (BATF3, r = -0.181) and type-I anti-tumor responses (CD8A, r = -0.173 and GZMB, r = -0.171). Furthermore, our results showed that high SQLE expression indicated low CTL infiltration and poor OS in PAAD patients (**Figure 8D**, p < 0.05). These results suggest that high SQLE expression predicted depletion of cytotoxic lymphocytes and loss of anti-tumor ability, leading to unfavorable responses to immunotherapy.

## Discussion

Presently, the prognosis of PAAD remains poor despite radical resection, mainly because of the lack of effective adjuvant therapy; therefore, the development of effective target biomarkers or promising drugs is urgently needed. Previous studies have demonstrated that SQLE promotes oncogenesis and metastasis in multiple human cancers by regulating cholesterol metabolism. However, a comprehensive understanding of SQLE in PAAD remained to be achieved.

In this study, we first performed pan-cancer analysis of SQLE expression and demonstrated that SQLE is highly expressed in PAAD. Survival and gene alteration analyses suggested that high expression and alteration of SQLE predicted the grim prognosis of PAAD, including OS, DFS, DSS, and PFS. miRNAs can modulate target gene expression through complex regulatory networks (41, 42). Therefore, it is essential to identify upstream miRNAs that participate in regulating SQLE expression. Twenty-one miRNAs were identified as pivotal regulators of SQLE. Among them, miR-194-5p was considered to potentiate the survival of tumor-repopulating cells, leading to radiotherapy failure in PAAD (43). Interestingly, microRNA-205, as a tumor suppressor, could re-sensitize gemcitabine-resistant pancreatic cancer cells and reduce the proliferation of cancer stem cells and tumor growth in mouse models (44). In addition, miR-92a-3p promotes EMT progression and metastasis by inhibiting PTEN and activating Akt/Snail signaling in hepatocellular carcinoma (45). miR-429 can be inhibited by an X-inactive specific transcript and upregulate the expression of ZEB1 to promote migration and invasion in PAAD (46).

After a comprehensive analysis of these 21 miRNAs in PAAD, including expression and survival analyses, miR-363-3p was recognized as the most potential upstream regulator of SQLE. Reportedly, miR-363-3p may play a crucial role in the progression of ovarian cancer (47). However, the role and function of miR-363-3p in PAAD have not been previously reported. We therefore speculate that miR-363-3p is involved in the pathological processes of PAAD by regulating SQLE function.

Immune cell infiltration into the tumor microenvironment is closely related to the therapeutic efficiency and prognosis of multiple human cancers, including gastric cancer (48) and colorectal cancer (49–51). Different immune cell-infiltrating subsets in the PAAD microenvironment were considered as independent prognostic characteristic factors (52). Furthermore, single-cell transcriptomics of PAAD indicated substantial immunological heterogeneities and T cell infiltration differences in the microenvironment. (53, 54). Our results emphasized that SQLE expression is negatively correlated with the infiltration of CD4+ T cells and NK cells, whereas it is positively correlated with the infiltration of CD8+ T cells and neutrophils in PAAD. Our findings suggest that SQLE may regulate the immune microenvironment in PAAD.

Immune checkpoint inhibitors comprise the most promising strategy for treating solid tumors (55), especially targeting PD-1 and CTLA4. However, the PD-1/PD-L1 blockade has proven to have limited effectiveness in PAAD (56). Therefore, we evaluated the relationship between SQLE expression and tumor immune biomarkers to identify new therapeutic strategies. Our results showed that SQLE expression was negatively correlated with PDCD1, LAG3, CTLA4, and CD160 expression, suggesting that the combined application of the SQLE inhibitor terbinafine and immune checkpoint blockade may improve the efficacy of PAAD. Moreover, the relation between SQLE expression and tumor immune feature was always consistent with the copy number of SQLE, whereas contrary to the results of SQLE methylation. We hypothesized that the methylation may inhibit the expression of SQLE, and thus caused these results.

We hypothesized that SQLE regulates the immune microenvironment through metabolic pathways. Enrichment analysis showed that SQLE-related partners are involved in cholesterol and lipid metabolism. KEGG and GO analyses suggested that “metabolic pathways” and “steroid biosynthesis” are associated with the function of SQLE in carcinogenesis. Yang et al. reported that cholesterol metabolism affects CD8+ T lymphocyte function (39). Cholesterol homeostasis is regulated by SCAP-SREBP2 and is essential for macrophage function (57). Moreover, statin use, by inhibiting cholesterol biosynthesis, could reduce mortality risk and improve survival of patients with PAAD (58). Finally, SQLE alteration was associated with high TMB, and its expression is negatively correlated with the infiltration of CTLs in melanoma and PAAD, leading to poor outcome of immunotherapy. Although the correlation between TMB and outcome of immunotherapy for PAAD has not been adequately elucidated, the lack of CTLs appears to underlie the ineffectiveness of immunotherapy in PAAD (59–61). Preclinical mouse models have suggested that increasing the infiltration of CTLs could improve the efficiency of checkpoint blockade in PAAD (62). In summary, our results indicate that SQLE influences the immune microenvironment and immunotherapy outcomes in patients with PAAD. Immunotherapy based on metabolic intervention may be a novel approach in treating PAAD, and interdisciplinary combination therapy may help overcome the bottleneck of cancer treatment.

Taken together, we demonstrate that SQLE expression is upregulated in multiple types of human cancer (including PAAD) and negatively correlated with the prognosis of PAAD. We also report an upstream miRNA, miR-363-3p, as a key regulator of SQLE expression in PAAD. SQLE could regulate the infiltration of tumor immune cells and the expression of immune checkpoints. SQLE plays a crucial role in cholesterol metabolism, and high SQLE expression is associated with poor immunotherapy outcomes. SQLE blockade may improve the efficiency of PAAD immunotherapy. Nevertheless, these results should be validated through additional wet experiments and clinical trials in the future.

## Data Availability Statement

The datasets presented in this study can be found in online repositories. The names of the repository/repositories and accession number(s) can be found in the article/**Supplementary Material**.

## Author Contributions

XW designed this study. WY, YC, ZC, Z-pH, and PH performed bioinformatic analyses. WY performed the wet experiments and wrote the manuscript. JK revised the manuscript. All authors have read the final version of this manuscript.

## Funding

This study was supported by the National Key R&D Program of China (No. 2017YFC1308800), National Key Clinical Discipline, National Natural Science Foundation of China (No. 81972212, No. 82003197), Guangdong Natural Science Foundation (No. 2019A1515010063), Science and Technology Planning Project of Guangdong Province, China (No. 2021A0505030028), Science and Technology Planning Project of Guangzhou City (No. 202102020186), the program of Guangdong Provincial Clinical Research Center for Digestive Diseases (2020B1111170004), Postdoctoral Fund project of The Sixth Affiliated Hospital, Sun Yat-sen University (No. R20210217202102987), and Youth Fund for Basic and Applied Basic Research of Guangdong Province (No. 2021A1515111128).

## Conflict of Interest

The authors declare that the research was conducted in the absence of any commercial or financial relationships that could be construed as a potential conflict of interest.

## Publisher’s Note

All claims expressed in this article are solely those of the authors and do not necessarily represent those of their affiliated organizations, or those of the publisher, the editors and the reviewers. Any product that may be evaluated in this article, or claim that may be made by its manufacturer, is not guaranteed or endorsed by the publisher.

## Supplementary Material

Supplementary Figure 1 Disease-free survival (DFS) analysis for SQLE in multiple human cancers. **(A–G)** DFS curves of SQLE in ACC **(A)**, BLCA **(B)**, HNSC **(C)**, LUSC **(D)**, PAAD **(E)**, SARC **(F)**, and UVM **(G)**.

Supplementary Figure 2 SQLE expression, copy number, and methylation are associated with tumor immune features. **(A–D)** Correlation of SQLE with lymphocyte **(A)**, immuno-inhibitor **(B)**, MHC molecule **(C)**, and immunostimulator **(D)**.

## Abbreviations

PAAD, pancreatic adenocarcinoma; SQLE, squalene epoxidase; OS, overall survival; DFS, disease-free survival; DSS, disease-specific survival; PFS, progression-free survival; TIME, tumor immune microenvironment; BLCA, bladder urothelial carcinoma; BRCA, breast invasive carcinoma; CESC, cervical squamous cell carcinoma and endocervical adenocarcinoma; COAD, colon adenocarcinoma; ESCA, esophageal carcinoma; HNSC, head and neck squamous cell carcinoma; LIHC, liver hepatocellular carcinoma; LUSC, lung squamous cell carcinoma, READ, rectal adenocarcinoma; STAD, stomach adenocarcinoma; UCEC, uterine corpus endometrial carcinoma; KIRP, kidney renal papillary cell carcinoma; PRAD, prostate adenocarcinoma; THCA, thyroid carcinoma; ACC, adrenocortical carcinoma; DLBC, lymphoid neoplasm diffuse large B-cell lymphoma; OV, ovarian serous cystadenocarcinoma; THYM, thymoma; UCS, uterine carcinosarcoma; LGG, lower grade glioma, SKCM, skin cutaneous melanoma; TGCT, testicular germ cell tumors; PDCD1, programmed cell death protein 1; LAG3, lymphocyte activation gene 3; CTLA-4, cytotoxic T-lymphocyte-associated protein 4; TMB, tumor mutation burden; CTL, cytotoxic T lymphocyte.
