# Supplementary material for: SQLE, A Key Enzyme in Cholesterol Metabolism, Correlates With Tumor Immune Infiltration and Immunotherapy Outcome of Pancreatic Adenocarcinoma
Source: Front Immunol. 2022 May 26;13:864244. doi: 10.3389/fimmu.2022.864244 (PMC9204319; doi:10.3389/fimmu.2022.864244)
Supplement: Supplementary file 1 [file DataSheet_1.docx]

Supplementary Table 1 The annotation for X-axis in figure 7N.

| GO terms | Annotation |
| --- | --- |
| GO:0055114 | oxidation-reduction process |
| GO:0006695 | cholesterol biosynthetic process |
| GO:0006629 | lipid metabolic process |
| GO:0000209 | protein polyubiquitination |
| GO:0008299 | isoprenoid biosynthetic process |
| GO:0016125 | sterol metabolic process |
| GO:0006633 | fatty acid biosynthetic process |
| GO:0043161 | proteasome-mediated ubiquitin-dependent protein catabolic process |
| GO:0016126 | sterol biosynthetic process |
| GO:0000266 | mitochondrial fission |
| GO:0006521 | regulation of cellular amino acid metabolic process |
| GO:0008203 | cholesterol metabolic process |
| GO:0043488 | regulation of mRNA stability |
| GO:0090263 | positive regulation of canonical Wnt signaling pathway |
| GO:0006897 | endocytosis |
| GO:0090090 | negative regulation of canonical Wnt signaling pathway |
| GO:0033490 | cholesterol biosynthetic process via lathosterol |
| GO:0036101 | leukotriene B4 catabolic process |
| GO:0033489 | cholesterol biosynthetic process via desmosterol |
| GO:0003374 | dynamin polymerization involved in mitochondrial fission |
| GO:0006690 | icosanoid metabolic process |
| GO:0061025 | membrane fusion |
| GO:0002479 | antigen processing and presentation of exogenous peptide antigen via MHC class I, TAP-dependent |
| GO:0006303 | double-strand break repair via nonhomologous end joining |
| GO:0038061 | NIK/NF-kappaB signaling |
| GO:0051437 | positive regulation of ubiquitin-protein ligase activity involved in regulation of mitotic cell cycle transition |
| GO:0031145 | anaphase-promoting complex-dependent catabolic process |
| GO:0006368 | transcription elongation from RNA polymerase II promoter |
| GO:0060071 | Wnt signaling pathway, planar cell polarity pathway |
| GO:0002223 | stimulatory C-type lectin receptor signaling pathway |
| GO:0009615 | response to virus |
| GO:0033209 | tumor necrosis factor-mediated signaling pathway |
| GO:0050821 | protein stabilization |
| GO:0000086 | G2/M transition of mitotic cell cycle |
| GO:0003095 | pressure natriuresis |
| GO:0034653 | retinoic acid catabolic process |
| GO:0006766 | vitamin metabolic process |
| GO:0048387 | negative regulation of retinoic acid receptor signaling pathway |
| GO:0055078 | sodium ion homeostasis |
| GO:0036503 | ERAD pathway |
| GO:0006691 | leukotriene metabolic process |
| GO:0006893 | Golgi to plasma membrane transport |
| GO:0019373 | epoxygenase P450 pathway |
| GO:0006699 | bile acid biosynthetic process |
| GO:0001676 | long-chain fatty acid metabolic process |
| GO:0019369 | arachidonic acid metabolic process |
| GO:0061024 | membrane organization |
| GO:0003091 | renal water homeostasis |
| GO:0031648 | protein destabilization |
| GO:0006986 | response to unfolded protein |
| GO:0031623 | receptor internalization |
| GO:0006631 | fatty acid metabolic process |
| GO:0006606 | protein import into nucleus |
| GO:0032092 | positive regulation of protein binding |
| GO:0032305 | positive regulation of icosanoid secretion |
| GO:0048285 | organelle fission |
| GO:0006696 | ergosterol biosynthetic process |
| GO:0042377 | vitamin K catabolic process |
| GO:0042361 | menaquinone catabolic process |
| GO:0042360 | vitamin E metabolic process |
| GO:0042376 | phylloquinone catabolic process |
| GO:0006705 | mineralocorticoid biosynthetic process |
| GO:0046602 | regulation of mitotic centrosome separation |
| GO:0035754 | B cell chemotaxis |
| GO:0070508 | cholesterol import |
| GO:0051988 | regulation of attachment of spindle microtubules to kinetochore |
| GO:0061179 | negative regulation of insulin secretion involved in cellular response to glucose stimulus |
| GO:0045839 | negative regulation of mitotic nuclear division |
| GO:0006704 | glucocorticoid biosynthetic process |
| GO:0097267 | omega-hydroxylase P450 pathway |
| GO:2000188 | regulation of cholesterol homeostasis |
| GO:0046621 | negative regulation of organ growth |
| GO:0051085 | chaperone mediated protein folding requiring cofactor |
| GO:0001682 | tRNA 5'-leader removal |
| GO:0006703 | estrogen biosynthetic process |
| GO:0006702 | androgen biosynthetic process |
| GO:0002031 | G-protein coupled receptor internalization |
| GO:0016021 | integral component of membrane |
| GO:0005789 | endoplasmic reticulum membrane |
| GO:0005829 | cytosol |
| GO:0016020 | membrane |
| GO:0005654 | nucleoplasm |
| GO:0005783 | endoplasmic reticulum |
| GO:0031090 | organelle membrane |
| GO:0005794 | Golgi apparatus |
| GO:0005813 | centrosome |
| GO:0043231 | intracellular membrane-bounded organelle |
| GO:0005874 | microtubule |
| GO:0000785 | chromatin |
| GO:0031966 | mitochondrial membrane |
| GO:0030176 | integral component of endoplasmic reticulum membrane |
| GO:0030496 | midbody |
| GO:0005905 | clathrin-coated pit |
| GO:0000502 | proteasome complex |
| GO:0005643 | nuclear pore |
| GO:0001917 | photoreceptor inner segment |
| GO:0005811 | lipid particle |
| GO:0030868 | smooth endoplasmic reticulum membrane |
| GO:0005655 | nucleolar ribonuclease P complex |
| GO:0008541 | proteasome regulatory particle, lid subcomplex |
| GO:0031597 | cytosolic proteasome complex |
| GO:0036513 | Derlin-1 retrotranslocation complex |
| GO:0005838 | proteasome regulatory particle |
| GO:0005506 | iron ion binding |
| GO:0020037 | heme binding |
| GO:0044822 | poly(A) RNA binding |
| GO:0016705 | oxidoreductase activity, acting on paired donors, with incorporation or reduction of molecular oxygen |
| GO:0004497 | monooxygenase activity |
| GO:0003924 | GTPase activity |
| GO:0005525 | GTP binding |
| GO:0019825 | oxygen binding |
| GO:0008017 | microtubule binding |
| GO:0003854 | 3-beta-hydroxy-delta5-steroid dehydrogenase activity |
| GO:0070330 | aromatase activity |
| GO:0031625 | ubiquitin protein ligase binding |
| GO:0004872 | receptor activity |
| GO:0050051 | leukotriene-B4 20-monooxygenase activity |
| GO:0018685 | alkane 1-monooxygenase activity |
| GO:0016628 | oxidoreductase activity, acting on the CH-CH group of donors, NAD or NADP as acceptor |
| GO:0016709 | oxidoreductase activity, acting on paired donors, with incorporation or reduction of molecular oxygen, NADPH as one donor, and incorporation of one atom of oxygen |
| GO:0016740 | transferase activity |
| GO:0004769 | steroid delta-isomerase activity |
| GO:0000254 | C-4 methylsterol oxidase activity |
| GO:0008401 | retinoic acid 4-hydroxylase activity |
| GO:0070628 | proteasome binding |
| GO:0050998 | nitric-oxide synthase binding |
| GO:0008392 | arachidonic acid epoxygenase activity |
| GO:0001972 | retinoic acid binding |
| GO:0008395 | steroid hydroxylase activity |
| GO:0050661 | NADP binding |
| GO:0019003 | GDP binding |
| GO:0097258 | 20-hydroxy-leukotriene B4 omega oxidase activity |
| GO:0000247 | C-8 sterol isomerase activity |
| GO:0052871 | alpha-tocopherol omega-hydroxylase activity |
| GO:0050613 | delta14-sterol reductase activity |
| GO:0097259 | 20-aldehyde-leukotriene B4 20-monooxygenase activity |
| GO:0031749 | D2 dopamine receptor binding |
| GO:0004526 | ribonuclease P activity |
| GO:1990381 | ubiquitin-specific protease binding |
