# Supplementary figures and images for: SQLE, A Key Enzyme in Cholesterol Metabolism, Correlates With Tumor Immune Infiltration and Immunotherapy Outcome of Pancreatic Adenocarcinoma
Source: Front Immunol. 2022 May 26;13:864244. doi: 10.3389/fimmu.2022.864244 (PMC9204319; doi:10.3389/fimmu.2022.864244)

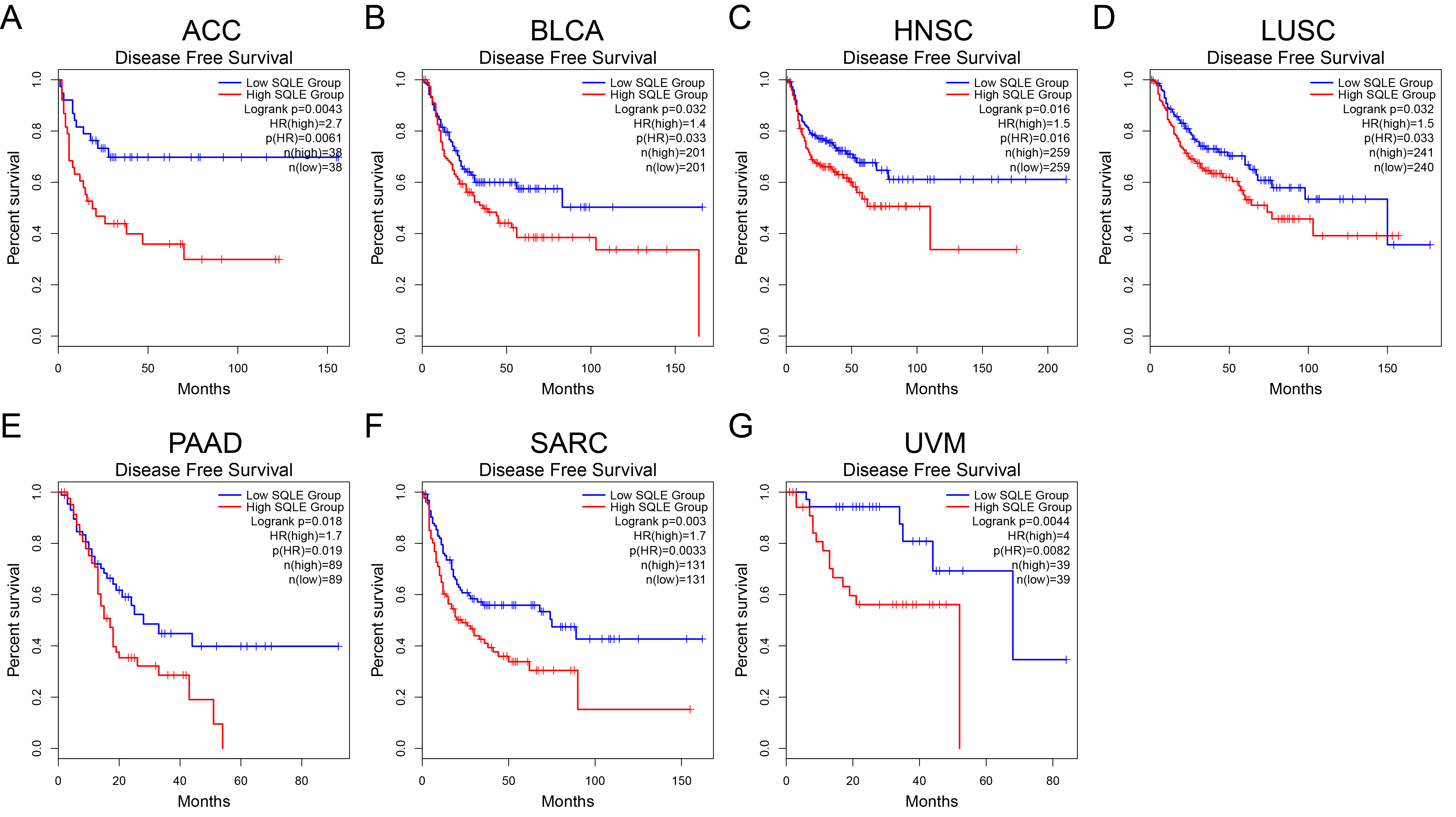

Supplement: Supplementary Figure 1 — Disease-free survival (DFS) analysis for SQLE in multiple human cancers. (A–G) DFS curves of SQLE in ACC (A), BLCA (B), HNSC (C), LUSC (D), PAAD (E), SARC (F), and UVM (G). [file Image_1.tif]

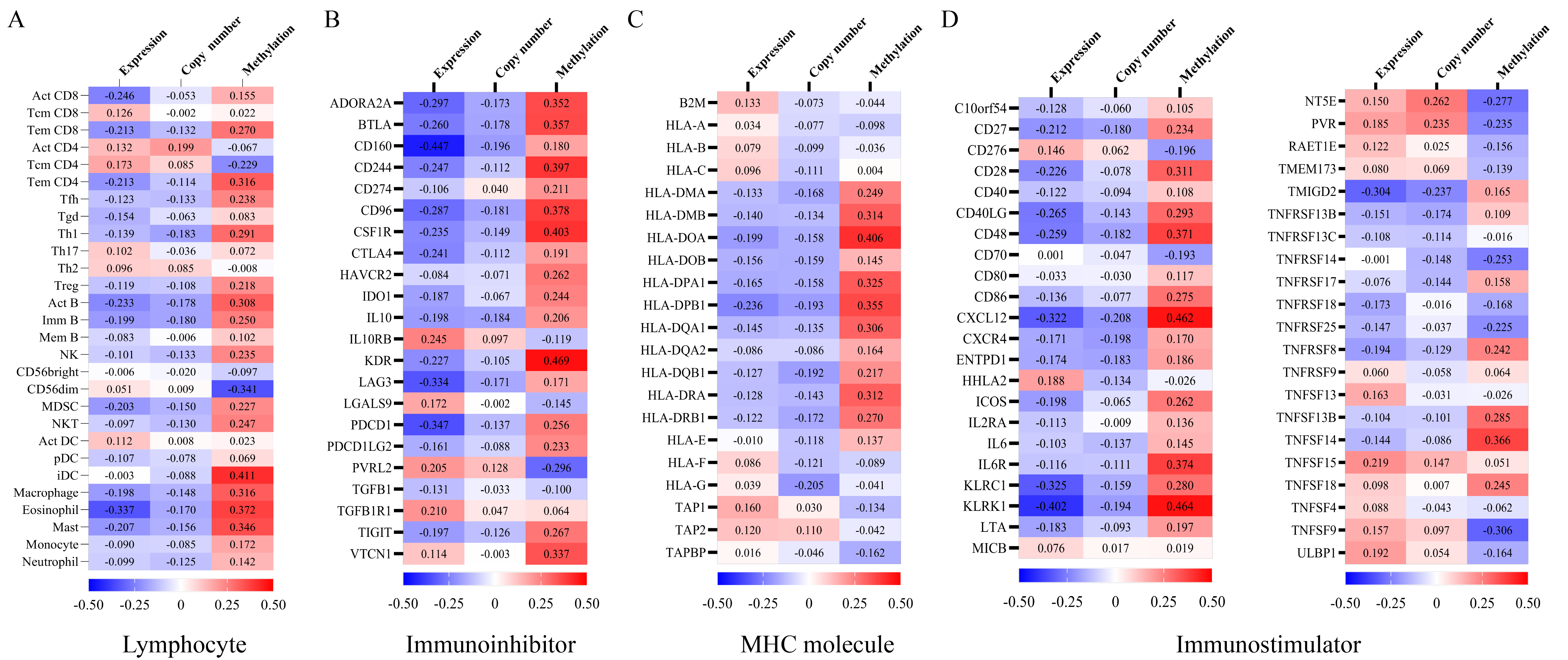

Supplement: Supplementary Figure 2 — SQLE expression, copy number, and methylation are associated with tumor immune features. (A–D) Correlation of SQLE with lymphocyte (A), immuno-inhibitor (B), MHC molecule (C), and immunostimulator (D). [file Image_2.tif]
